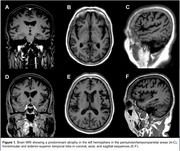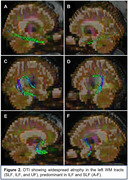# Atypical AD with mixed logopenic / non‐fluent primary progressive aphasia and ANXA11 gene mutation: a case report

**DOI:** 10.1002/alz70857_101641

**Published:** 2025-12-25

**Authors:** Paulo Eduardo Lahoz Fernandez, Camila Farias de Araujo, Rairis Barbosa Nascimento, Marcelo dos Santos Bandeira Filho, Gustavo Dalul Gomez, Alan Cronemberger Andrade, Sheilla de Medeiros, Correia Marin, Paulo Henrique Ferreira Bertolucci

**Affiliations:** ^1^ Federal University of São Paulo ‐ UNIFESP, São Paulo, SP, Brazil

## Abstract

**Background:**

Logopenic primary progressive aphasia (lvPPA) is an atypical language variant of Alzheimer's disease (AD). Non‐fluent / agrammatic (nfvPPA) is related to frontotemporal lobar degeneration (FTLD) but is rarely found in AD pathology. The annexin A11 (ANXA11) gene mutation is usually associated with amyotrophic lateral sclerosis / FTLD, but recently, it has been reported in the semantic variant (svPPA). Diffusion tensor tractography (DTI) can be used in PPA to localize white matter (WM) tract changes in the inferior longitudinal fasciculus (ILF), superior longitudinal fasciculus (SLF), and uncinate fasciculus (UF). The nfaPPA WM pattern is seen in the left SLF; sv‐PPA reveals focal severe left/bilateral changes in UF and anterior ILF; the lvPPA pattern is more widespread in the left FLS, FLI, and UF, marked in the middle/posterior ILF.

**Method:**

We report the clinical, genetic, and imaging features of a rare case of atypical AD with mixed PPA (lvPPA / nfvPPA) phenotype and ANXA11 gene mutation.

**Result:**

A right‐handed 65‐year‐old woman with 8 years of a language progressive impairment with difficulty in finding words for expression, repeating sentences, and naming objects, which affected her daily activities, scoring 14/30 on MMSE, 3 on semantic verbal fluency, and 10/20 on the Boston Naming Test. She also had memory problems, such as forgetting recent events/appointments and losing objects, and over the years, manifested effortful speech and agrammatism. She was illiterate, her medical family history was negative, and the neurological exam was unremarkable. The CSF indicated increased t‐tau/p‐tau levels and a decreased Aβ 42/40 ratio, suggesting an AD pathology. The genetic testing confirmed the ANXA11 gene mutation. MRI revealed a left‐predominant atrophy in the frontoinsular and perisylvian/temporoparietal areas. The DTI showed widespread WM atrophy in the left SLF, ILF, and UF tracts, predominant in ILF and SLF, consistent with this mixed PPA phenotype.

**Conclusion:**

This report can provide valuable insight into considering AD when confronted with atypical clinical presentations with mixed‐PPA, as in this case. It also reinforces the clinical variability of ANXA11 gene mutations and highlights the importance of using DTI for detecting WM matter‐specific pattern changes in different PPA phenotypes.